# Successful conservative treatment of intestinal perforation in VLBW and ELBW neonates: a single centre case series and review of the literature

**DOI:** 10.1186/s12887-019-1641-1

**Published:** 2019-07-25

**Authors:** Nan Ye, Yurong Yuan, Lei Xu, Riccardo E. Pfister, Chuanzhong Yang

**Affiliations:** 1 Department of Neonatology, Affiliated Shenzhen Maternity & Child Healthcare Hospital, Southern Medical University, Hong Li Road 2004, Futian District, Shenzhen, 518028 Guangdong China; 2 Department of Obstetrics, The University of Hong Kong-Shenzhen Hospital, Haiyuan 1st Road, Futain District, Shenzhen, 518053 Guangdong China; 3 Department of Paediatrics, Neonatology and Paediatric Intensive Care Services, University Hospitals of Geneva and Geneva University, Geneva, Switzerland; 4 NICU Neonatal Department, Affiliated Shenzhen Maternity & Child Healthcare Hospital, Southern Medical University, Chief’s office, 4th floor, Building 5, Hong Li Road 2004, Futian District, Shenzhen, 518028 Guangdong China

**Keywords:** Intestinal perforation, Pneumoperitoneum, Conservative treatment, VLBW, ELBW

## Abstract

**Background:**

The current standard treatment of neonates with intestinal perforation is surgery. However, the mortality rate after surgical treatment for intestinal perforation is very high for very low birth weight (VLBW) and extremely low birth weight (ELBW) neonates. In this review, conservative treatment of pneumoperitoneum among VLBW and ELBW neonates is investigated.

**Methods:**

Between January 2015 and December 2017, data from all of the VLBW and ELBW neonates with pneumoperitoneum who survived without surgical treatment were collected from Shenzhen Maternity and Child Healthcare Hospital in Guangdong, China. Twenty-two neonates with birth weight less than 1500 g were diagnosed with pneumoperitoneum. Following careful evaluation and discussion, eleven were treated conservatively and this was successful in eight. Details of the eight neonates including birth weight, gestational age, gender, risk factors, time of the perforation, treatment and prognosis were retrospectively recorded. A literature review was performed of previously reported cases that had used conservative treatment.

**Results:**

The median gestational age and birth weight of the eight neonates were 27^+ 1^ weeks (range 24w^+ 3^ to 31w^+ 6^) and 855 g (range 650 g to 1440 g), respectively. Pneumoperitoneum was confirmed by X-ray in all at a median of 8 days of life. They received full parenteral support for a median of 22 days. All eight neonates received a combination of piperacillin-tazobactam and meropenem as first-choice antibiotics, two of them also received fluconazole as anti-fungal medication. Median duration of hospitalisation was 80 days.

**Conclusions:**

Conservative treatment with careful surveillance may be a practical choice for the VLBW and ELBW neonates with intestinal perforation. Further studies are needed for confirmation.

## Background

Intestinal perforation is a severe complication that causes high mortality rates in preterm neonates and is usually characterized by abdominal distension and pneumoperitoneum on abdominal X-rays. The current standard treatment of neonates with intestinal perforation is surgery. However, while laparotomy may fix the lesion in very low birth weight (VLBW) neonates and extremely low birth weight (ELBW) neonates, it also has considerable risks, including anaesthesia, operative risks, and possible infections [1]. We noticed that some closely monitored VLBW/ELBW neonates with pneumoperitoneum may gain full recovery without surgical intervention or peritoneal drainage with appropriate nutrition and pharmacological strategies. This case series was undertaken to summarize the clinical experience in a small number of cases, and to discuss these in the light of the current literature.

## Methods

**Fig. 1:**
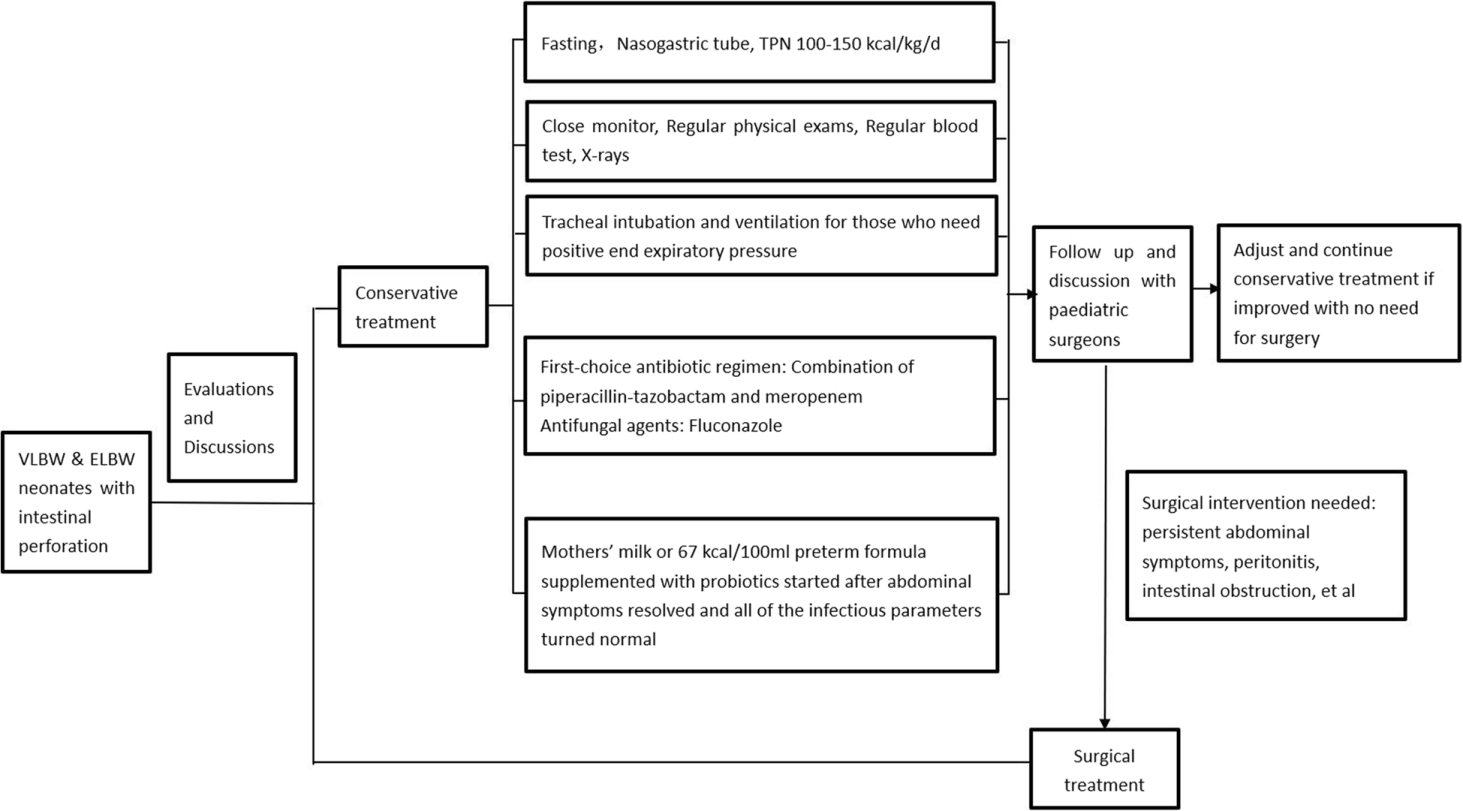
The management strategies of conservative treatment

After approval by the institutional medical ethics committee (SFYLS [2018] No.239), a retrospective study was conducted in neonatal intensive care unit (NICU) of Shenzhen Maternity and Child Healthcare Hospital in Guangdong, China. From January 2015 to December 2017, all preterm neonates of birth weight less than 1500 g diagnosed with intestinal perforation within 2 weeks of birth were reviewed, intestinal malformations were ruled out, and the cases that made a full recovery without surgical intervention were analysed further. For each of these cases, a detailed discussion was held between the neonatologists, the paediatric surgeons, and the parents. The advantages and risks of conservative treatment and surgery were weighed. With the full agreement and cooperation of the parents, cases with low-grade clinical symptoms were given conservative treatment with close monitoring. Low-grade clinical symptoms means stable general condition and low risk of peritonitis, and are characterized by: 1, Stable vital sign, no deterioration of heart rate, blood pressure and blood oxygen saturation under normal respiratory support; 2, Physical examination shows no rigidity of abdominal wall, no ‘blue colour’ on the abdominal wall; 3, Under X-ray, air inflation exists in intestine, no obstruction (dilatation or air-fluid level within intestine), no ascites, no pneumatosis intestinalis or portal venous gas, and no sign of intestinal malformation shown. The main management strategy is shown in Fig. 1. Antibiotics were used as soon as intestinal perforation was diagnosed. The total course of antibiotic therapy was at least 2 weeks. The first-choice antibiotics were downgraded once the infectious parameters (WBC and CRP) were normal, antibiotics were then stopped completely once patients were tolerating feeds (50-60 ml/kg/day).

**Fig. 2:**
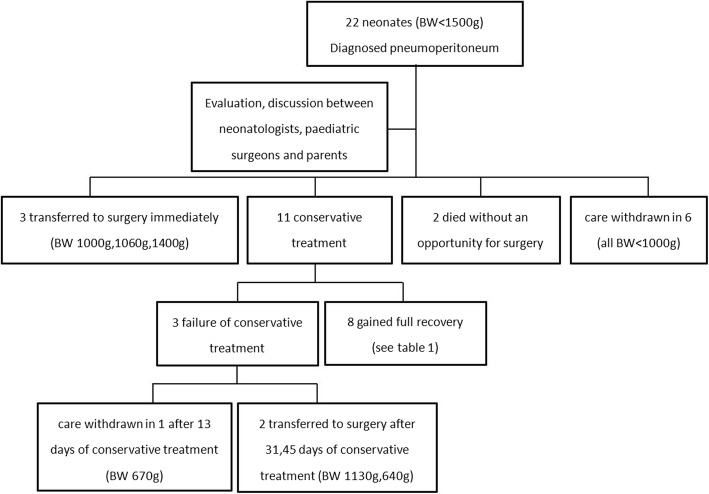
Flow chart of cases included

All of the neonates with birth weight (BW) ≥1500 g were transferred to surgery immediately on the diagnosis of intestinal perforation. From January 2015 to December 2017, 22 neonates with BW < 1500 g were diagnosed with pneumoperitoneum secondary to intestinal perforation. Three of them were immediately transferred to surgery (with a BW of 1000 g, 1060 g, 1400 g, respectively) after multidisciplinary consultation, two died of severe infection without an opportunity for surgery. Considering the great risks and potential severe complications after surgery, parents of six of the neonates (all BW < 1000 g) decided to stop all treatments after perforation was diagnosed. Eleven neonates received conservative treatment, eight of them made a full recovery, two of them were transferred to surgery because of incomplete intestinal obstruction, the other one (BW 670 g) developed IVH (grade III) and the parents decided to stop intensive care after thirteen day’s conservative treatment, died in the end [Fig. 2].

Details of the cases with conservative treatment were reviewed and analysed. These details included maternal complications, antenatal infections (defined as high infectious risk factor and abnormally elevated white blood cell/C-reactive protein (WBC/CRP) within 48 h of delivery), antenatal corticosteroids, and neonatal parameters such as gestational age (GA), birth weight (BW), surfactant, ibuprofen, respiratory support, laboratory tests results, treatments, and complications. The time to achieve full enteral feedings and hospital discharge were recorded. The clinical features of the neonates who underwent surgery were also briefly summarized as comparation. Descriptive statistics were used to summarize the data, and are presented as percentiles, medians, and ranges.

## Results

**Fig. 3:**
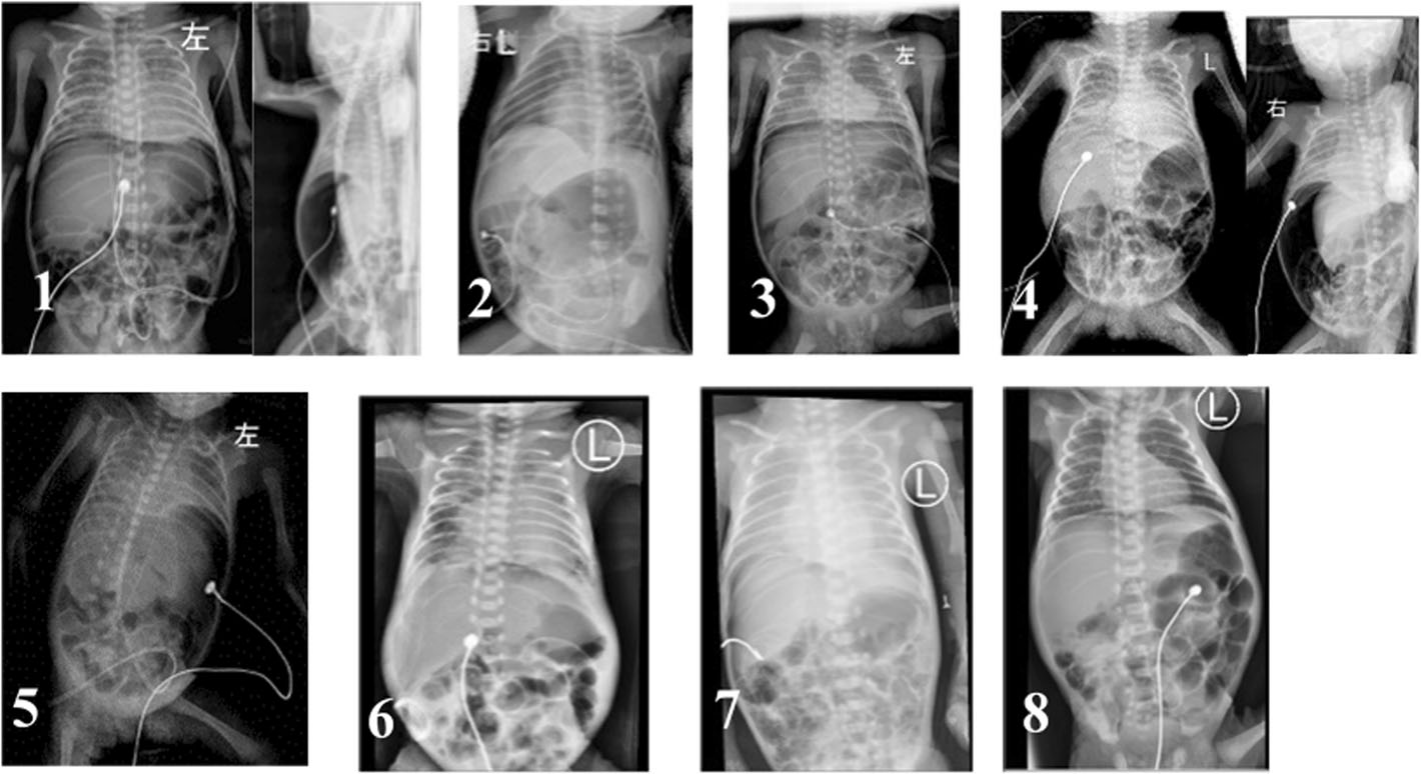
Diagnostic X-rays of pneumoperitoneum in all 8 cases

The diagnosis of intestinal perforation was made at a median of 8 days of life. Abdominal X-rays revealed the pneumoperitoneum [Fig. 3] (X-rays are indicated whenever there were abnormal abdominal findings or whenever catheterization was done). Data from the eight cases, including demographics, medical history, and clinical course, were summarized in Table 1.

**Table 1:** Demographics and follow-up of case series

	Case1	Case2	Case3	Case4	Case5	Case6	Case7	Case8	Median/Percentage
Gender	M	F	F	F	F	F	M	M	
GA(W)	27^+ 2^	31^+ 6^	26^+ 6^	24^+ 6^	25^+ 5^	24^+ 3^	29^+ 4^	29^+ 1^	27^+ 1^
BW(g)	800	1440	910	690	700	650	1160	1300	855
Antenatal steroid	–	+	–	–	–	–	+	+	38%
Max Milk intake (ml/kg/d)	9	5.5	16.8	5.8	28.5	12.3	6.9	12.3	10.7
Antenatal infection	+	+	+	+	–	–	–	–	50%
Vaginal/CS	V	CS	V	V	V	V	CS	V	CS 25%
Asphyxia at birth	+	+	+	+	+	+	+	–	88%
Res support	nIMV	Hiflow	nIMV	nIMV	nIMV	nIMV	SIMV	Hiflow	
hsPDA	+	–	+	+	–	+	–	+	63%
ibuprofen	+	–	+	+	–	+	–	–	50%
Antibiotics used^a^	PT + M	PT + M	PT + M	PT + M + F	PT + M	PT + M	PT + M	PT + M + F	
WBC(×10^9/l)	24.9	13.2	44.1	29.0	19.5	38.9	6.6	38.9	
Neutrophil (%)	54.4	64.9	69.5	49.1	75.9	68.5	34.5	72.1	
CRP	< 0.5	75.7	< 0.5	1.2	0.9	35.3	2.1	9.9	
Postnatal day of discovering perforation	8	5	8	10	7	10	5	6	8
X-ray normalisation after discovery (day)	10	13	12	7	17	6	7	5	9
Postnatal day of start feeding	31	25	28	40	36	47	26	26	30
Fasting time	23	20	20	30	29	37	21	20	22
Postnatal day of full feed	60	50	68	155	73	149	50	58	64
Postnatal day of discharge	90	53	69	178	105	153	52	59	80

^a^*PT* Piperacillin-tazobactam, *M* Meropenem, *F* Fluconazole

In terms of maternity history, severe preeclampsia was present in cases 2 and 7, threatened labour in case 8, and an inevitable abortion (considered for pregnancies < 28 weeks) was seen in the other five cases. Only three cases (38%) received full antenatal corticosteroids. Two cases (25%) were born by caesarean section and four (50%) had antenatal infections. The median gestational age was 27^+ 1^ weeks (range 24w^+ 3^ to 31w^+ 6^), and the median birth weight was 855 g (range 650 g to 1440 g).

Seven of the neonates had a low 1-min Apgar score (< 7) at birth, and all received surfactant after birth. In the first 24 h after birth, two neonates had an episode of metabolic acidosis, and another two experienced combined respiratory and metabolic acidosis. Five neonates had a hemodynamically significant patent ductus arteriosus (hsPDA) and, in four of them oral ibuprofen was used for PDA closure. One case with hsPDA did not receive ibuprofen because of the perforation. Finally, PDA was still observed in one case with oral ibuprofen and later was closed by surgery.

In terms of respiratory support, two neonates used a high-flow nasal cannula, five neonates were on non-invasive mechanical ventilation (nIMV), and one neonate used synchronized intermittent mandatory ventilation (SIMV).

With regard to feeding, all the neonates were receiving breast milk through gastric tubes before perforation. The median maximum feeding volume at diagnosis for all of the eight neonates was 10.7 ml/k/d (5.5 to 28.5 ml/kg/d). Bile-stained gastric residues were found in one case (13%), indicating possible obstruction in intestine. Feeding intolerance (milk residues) was seen in three neonates (38%).

Abdominal distension was present in seven cases (88%). One neonate did not show any abdominal symptoms. In all eight cases, no erythema, tenderness or palpable lump and no bloody stool were seen. Upper gastrointestinal bleeding was observed in five neonates (63%), with dark red aspirates in the gastric tube.

Laboratory investigations within 24 h of the perforation showed elevated WBC/CRP in seven cases (88%). For all of the cases, liver and renal function parameters, as well as electrolytes, were within the normal range. Two neonates had hyperglycaemia and one had thrombocytopenia.

Due to the discovery of the pneumoperitoneum, all eight patients were closely monitored in the neonatal intensive care unit, with regular physical examinations and frequent abdominal girth measurements to detect any deterioration. Routine blood tests were done daily at the beginning (mostly first three days after the diagnosis of perforation), with blood cultures, and X-rays performed as required, primarily once per week. Follow up was performed in collaboration with the paediatric surgeons. A nasogastric tube was placed in all of the neonates for gastric decompression and fasting started immediately. After the perforation, five neonates were reintubated to avoid intestinal gas pressure from the continuous positive airway pressure. All of the neonates received total parenteral nutrition (TPN) with 100–150 kcal/kg/d and essential electrolytes. Selected microelements were given weekly during the exclusive parenteral nutrition. A combination of piperacillin-tazobactam and meropenem was the first-choice antibiotic regimen upon discovery of the pneumoperitoneum. Fluconazole was added in two cases who were at high risk of fungal infection. Two patients received intravenous immunoglobulin (IVIG) therapy as an adjuvant anti-infective therapy since there was no significant improvements in their infectious parameters (WBC or CRP) in the first three days. Five patients with pneumoperitoneum lasting more than 7 days were treated with fresh frozen plasma to improve immunity, provide additional support for coagulation function, and promote intestinal wall healing. Dopamine was used in all of the cases at 5 μg/kg/min to improve microcirculation and maintain hemodynamic stability during the first few days.

None of the eight cases had a positive blood culture result. The disappearance of free intraperitoneal gas in X-rays took place at a median of 9 days (range 5–17 days) after diagnosis. Median fasting time was 22 days (range 20–37 days). Mothers’ milk or 67 kcal/100 ml preterm formula supplemented with probiotics was introduced after the disappearance of abdominal symptoms and normalisation of infectious parameters (including WBC and CRP). Full enteral feeds were established in all eight patients. In case 8, bloody stool was observed once when the feeding volume reached 50 ml/kg/d, but the patient tolerated feedings after a change to hydrolysed protein formula. Case 3 was a patient with congenital heart disease. She had pulmonary valve stenosis and low-grade insufficiency and was transferred after reaching full feedings to the cardiac surgery department for further treatment. Seven patients were discharged home. The median time of hospitalisation was 80 days (range 52–178 days). Intraventricular haemorrhage (IVH) was present in case 4 (grade II) and case 7 (grade I). Retinopathy of prematurity (ROP) (stage II and III) was diagnosed in two of the cases.

The clinical features of the neonates who underwent surgery were briefly summarized in Table 2. The patient in case A was diagnosed as necrotizing enterocolitis (NEC) on day 39, which was consistent with pathological findings. Her parents decided to stop treatment because of large area of necrosis in her intestine and the worries of poor prognosis. In case B and C, the occurrence of intestinal perforation was early (both on day 3). Their parents chose an active surgical treatment and the operations were successful, the children grew up well during the follow-up. In case D and E, conservative treatment was tried for 22 and 37 days respectively, then they developed recurrent abdominal distension and were transferred to surgery with the diagnosis of incomplete intestinal obstruction. At the time of operation, their body weight reached 1500 g, 1170 g respectively, and the operations went well and both of them grew up well during the follow-up.

**Table 2:** Clinical features of the neonates underwent surgery

	Case A	Case B	Case C	Case D	Case E
Gender	F	M	F	M	F
GA(W)	27^+ 2^	27	30^+ 3^	30	24^+ 3^
BW(g)	1000	1060	1400	1130	640
Antenatal steroid	+	+	–	+	+
Max Milk intake (ml/kg/d)	15.3	0.5	1.5	4	1
Antenatal infection	+	+	–	–	–
Vaginal/CS	V	V	CS	CS	V
Asphyxia at birth	–	–	–	–	–
Res support	nIMV	nCPAP	Hiflow	nIMV	SIMV
WBC(×10^9/l)	14.4	11.5	9.4	12.6	38.3
Neutrophil (%)	62.5	65.6	35.2	49	71.3
CRP (mg/L)	175	0.5	< 1	< 0.5	3.03
Postnatal day of discovering perforation	39	3	3	9	8
Postnatal day of transferring to surgery	39	3	3	31	45
Pathology	NEC	SIP	GP	Intestinal obstruction after perforation	Intestinal obstruction after perforation
Result of surgery	Abandoned treatment during operation	improved	improved	improved	improved

## Discussion

NEC, spontaneous intestinal perforation (SIP), and gastric perforation (GP) are the most common causes of intestinal perforation in the premature neonates [2]. All of the reported cases in this study had clinical manifestations and X-ray confirmation of intestinal perforation. However, clinical symptoms of NEC, SIP and GP, such as abdominal distension, feeding intolerance, intestinal bleeding and infectious parameters are non specific. The pathogenesis of NEC is based on mucosal injury with subsequent bacterial translocation across the intestinal epithelial layer and deregulation of the innate immune defence leading to subsequent inflammation and tissue necrosis. In contrast, SIP and GP mainly affects infants with extremely low birth weights at early postnatal ages. These are characterized by an isolated perforation without surrounding necrosis or neutrophil infiltrate, and are often accompanied by a focal thinning or absence of the intestinal muscularis propria [3]. Some studies have differentiated between NEC and SIP based on clinical findings before surgery [3, 4]. In this study’s reported eight cases, since no surgeries were performed, no pathological samples confirmed the exact cause and location of the perforations. Due to the low-grade symptomatology and favourable clinical course, it was hypothesized that SIP/GP was the likely cause of perforations in this small cohort.

Survival of preterm infants has dramatically improved over the last decades [5]. However, the mortality rate after surgical treatment for intestinal perforation remains very high in extremely premature infants, especially in neonates who are at the lowest limit of viability [1]. Therefore, some authors have recommended peritoneal drainage (PD) as an initial treatment approach for NEC or SIP [6, 7], and they have found higher survival rates compared with laparotomy. This suggests that in certain situations, intestinal perforation may heal without laparotomy.

Gummalla [8] reported on a 560-g male baby, born at 23 + 6 weeks of gestational age who developed pneumoperitoneum secondary to pneumothorax on the 19th day of life. This author warned that pneumoperitoneum may not always indicate intestinal perforation that may require laparotomy. He suggested three other possible causes: secondary to cardiopulmonary resuscitation, mechanical ventilation, and pneumatosis cystoides intestinalis. These three potential causes were ruled out in all of the eight cases examined in this study. These causes should always be ruled out before laparotomy is performed.

Pneumoperitoneum is widely accepted as needing surgical intervention. In a retrospective 10-year report of 27 neonates with gastrointestinal perforations, the three neonates who did not undergo surgery all died [9]. Sawicka evaluated surgical treatment in a group of VLBW and ELBW neonates. There were 101 neonates treated between 2000 and 2009 who were included in this study from the Department of Paediatric Surgery. Their birth weights ranged from 450 g to 1500 g (mean of 952 g), and gestational ages ranged from 23 weeks to 32 weeks (mean of 27 weeks). Their diseases included NEC (28 patients), SIP (32), GP (4), congenital defects (31), and other diseases. Of 64 neonates with bowel perforations, 20 died (31%). In total, 30 patients died. Twenty-one of them were ELBW neonates. These results also confirm the significant mortality difference between NEC (65%) and SIP (19.5%) [10].

A study from the Thames Valley & Wessex Neonatal Network reviewed 381 infants born at less than 26 completed weeks of gestation [2]. Between April 2007 and March 2015, emergency laparotomy was indicated for 61 infants and performed in 57 of them. Four infants that had indications for laparotomy and severe comorbidity had intensive care withdrawn without surgery. Nine infants (16%) required more than one laparotomy. Fifteen infants required surgical patent ductus arteriosus ligation following laparotomy, and 17 had laser therapy for retinopathy due to prematurity. Overall, 42 infants with indications for laparotomy (69%) survived until discharge.

Pneumoperitoneum causes high mortality rates, even with surgical treatment. In contrast, Rizwan Ahmad Khan [11] reported on two infants with pneumoperitoneum (one was full term, the other was born at 34 weeks) were clinicians opted for a trial of conservative management. They did not find clinical features suggestive of NEC, and the infants recovered quickly and were discharged on the 10th and 11th postnatal day. The authors suggested SIP as the cause of the pneumoperitoneum, and therefore concluded that surgery may not always be necessary for SIP. Rahul Gupta [12] reported on a 36-week, 2.1-kg preterm infants that developed pneumoperitoneum on the second postnatal day. The infant had abdominal distension and delayed passage of meconium. Laboratory investigations were within the normal range. With conservative treatment, full oral feeds were gradually achieved by the seventh postnatal day. Gupta also concluded that pneumoperitoneum is not an absolute indication for surgery, and an individualized approach is needed.

The authors of this paper agree that surgery may not be indicated in all cases of intestinal perforation, and a personalized approach should be utilized for each patient. In the past, the literature describing the conservative treatment of intestinal perforation in VLBW/ELBW neonates contains mainly isolated case reports and few clinical series. In the author’s assumption, under appropriate conservative treatment, there are two possible reasons for the recovery of the eight cases in this paper: first, in the early period after birth, the VLBW/ELBW neonates had received only a small amount of mother milk in the NICU, the number of pathogenic bacteria in the intestine is not large enough to cause necrosis on the intestinal wall, in that case the lesion of an isolated perforation may cure easily; second, digestive glands of these neonates were still immature, few digestive juices were secreted and fewer leaked into the peritoneal cavity, causing very little chemical damage, which may recover without the help of surgical lavage. The patients in this study’s cohort required much longer hospital times than those in the published case reports. The reasons for this may have been their smaller gestational ages and birth weights, as well as more severe presentations of abnormal infectious parameters in neonates with lower immune competence.

Detailed assessment should be made before conservative/surgical treatment is selected. The eight patients treated conservatively meet with the following clinical features: the diagnosis of perforation was made in an early postnatal period (at a median of 8 days of life); the amount of milk they had received was relatively small (most of them were still in early minimal feeding period); their symptoms were relatively mild, and consistent with the symptoms of SIP (as mentioned in the method section). All the patients with BW less than 1000 g chose conservative treatment as first choice. As for older patients, patients with NEC (like case A), obstructions (case D and E) and any other problems that cannot be solved conservatively, surgery must be used. Since the number of cases in this study was limited, and treatment strategies were influenced by doctors’ experiences and the parents’ wishes, the understanding of different clinical features between surgical and non-surgical patient was limited. More work should be done to recognize which neonates would benefit most from conservative treatment.

Based on the outcomes of this study, full recovery without surgery is possible with close monitoring and appropriate conservative treatment in VLBW/ELBW neonates diagnosed with intestinal perforation. Hemodynamic instability in surgery may lead to some severe complications such as intraventricular haemorrhage (IVH) in VLBW and ELBW neonates, especially in the first week after birth, which may result in poor outcomes [13]. Conservative treatment in this early period after life may reduce these complications, and it can alleviate the anxiety of parents as well as medical staffs. In case D and E, incomplete intestinal obstruction occurred and conservative treatment failed. The failure might be due to lack of experience in the evaluation and treatment process. While since the surgery for case D and E succeeded and the outcome was satisfactory in the end, it can be hypothesized that for the neonates at the limits of viability, conservative treatment can also be a transitional therapy for intestinal perforation. Once the neonates gain weight and goes through the early unstable period, surgical operations will be safer.

Conservative treatment might also have drawbacks. Without laparotomy, intestinal perforation caused by congenital defects which cannot be confirmed by antenatal examinations or postnatal X-ray might be misdiagnosed. Emphasis should be placed on repetitive evaluation during conservative treatment so as not to delay the timing of surgery. In terms of anti-infection strategies, although antibiotics were downgraded and stopped as early as possible, the patients received 2~3 weeks of broad-spectrum antibiotics, which increased the risk of drug resistance. Doctors should strictly control the use of antibiotics, evaluate the pros and cons of conservative treatment periodically, and if necessary, transfer to surgery at appropriate time.

The study showed the feasibility of conservative treatment in VLBW/ELBW neonates with intestinal perforation, it still has a few limitations: firstly, it was a retrospective case series based on a small sample size, the patients who had care withdraw had to be excluded. Larger sample size and controlled studies may provide more evidence for the results. Ethical issues should be taken into consideration. Secondly, part of the clinical decisions were made from the willingness of parents and the doctors’ experience, which may result in practice variability. A clinical guide for the conservative treatment of intestinal perforation in VLBW and ELBW neonates is urgently needed. Furthermore, long-term outcomes of conservative treatment were not evaluated in the study, long-term follow-up are needed. The safety of conservative treatment, identification of beneficiary groups, and the timing of surgery should be the topics of further studies.

## Conclusions

It can be concluded that in VLBW and ELBW neonates with pneumoperitoneum suggestive of intestinal perforation, particularly in those presented with low-grade clinical symptoms (suspected of SIP), conservative treatment should be carefully considered as a first-choice therapeutic option in the early postnatal period. Under close monitoring and appropriate supportive treatment, full recovery can be expected, even with abnormal laboratory findings. Further studies are needed to supplement the evidence and guidelines are need for clinical treatment.

## Data Availability

The datasets used and/or analyzed during the current study are available from the corresponding author on reasonable request.

## Abbreviations

BW): Birth weight
ELBW): Extremely low birth weight
GA): Gestational age
GR): Gastric rupture/perforation
hsPDA): Hemodynamically significant patent ductusarteriosus
IVH): Intraventricular haemorrhage
IVIG): Immunoglobulin therapy
NEC): Necrotizing enterocolitis
nIMV): Non-invasive mechanical ventilation
ROP): Retinopathy of prematurity
SIMV): Synchronized intermittent mandatory ventilation
SIP): Spontaneous intestinal perforation
TPN): Total parenteral nutrition
VLBW): Very low birth weight
WBC/CRP): White blood cell/C-reactive protein
